# Burnout among dutch intensivists

**DOI:** 10.1186/2197-425X-3-S1-A140

**Published:** 2015-10-01

**Authors:** IA Meynaar, JLCM van Saase, T Feberwee, TM Aerts, J Bakker, W Thijsse

**Affiliations:** HagaZiekenhuis, ICU, Delft, Netherlands; Department of Internal Medicine, ErasmusMC University Medical Center, Rotterdam, Netherlands; Reinier de Graaf Hospital, Hospital Pharmacy, Delft, Netherlands; Department of Health Management, RET, Rotterdam, Netherlands; Department of Intensive Care Adults, ErasmusMC University Medical Center, Rotterdam, Netherlands

## Introduction

Burnout in healthcare workers is deleterious both for the patients as well as for the healthcare workers themselves with consequences ranging from less job satisfaction to increased mortality rates. Burnout in intensivists is reportedly high with almost half of French intensivists having burnout symptoms.

## Objectives

The present study was done to estimate incidence and prevalence of burnout in intensivists in the Netherlands and to identify risk factors for burnout.

## Methods

Two online questionnaires were sent: one to all intensivists in the Netherlands and one to the medical directors of Dutch Intensive Care Units (ICUs).

## Results

A reply was received of 318 out of 664 intensivists (47.9%). Results of 272 intensivists were evaluable, 12 of whom (4.4%) were diagnosed with burnout at the time of the questionnaire. No association was found between working conditions or personal characteristics and burnout, except for the association between burnout and conflict with the hospital management and between burnout and having complaints filed against one. From the medical directors questionnaire it was found that 7.4% of intensivists suffered from burnout in 2013.

## Conclusions

Incidence and prevalence of burnout among Dutch intensivist was found to be unexpectedly low as compared to the literature. No firm associations were found between burnout and working conditions or personal characteristics. The low incidence and prevalence of burnout in Dutch intensivists might be explained be a lower workload for intensivists as compared to the literature.

## Acknowledgement

This study was done with support of the Dutch Society for Intensive Care

